# Pre-emptive steroids for a severe oedematous Buruli ulcer lesion: a case report

**DOI:** 10.1186/s13256-015-0584-x

**Published:** 2015-05-01

**Authors:** Daniel P O’Brien, Sarah Huffam

**Affiliations:** 1 Department of Infectious Diseases, University Hospital, 292-392 Ryrie Street, Geelong, VIC 3220 Australia; 2 Department of Medicine and Infectious Diseases, Royal Melbourne Hospital, University of Melbourne, Grattan Street, Parkville, Melbourne, VIC 3052 Australia; 3 Manson Unit, Médecins Sans Frontières, 67-74 Saffron Street, London, EC1N 8QX UK

**Keywords:** Mycobacterium ulcerans, Buruli ulcer, Prednisolone, Treatment, Antibiotics, Oedematous

## Abstract

**Introduction:**

Severe oedematous forms of Buruli ulcer (BU) often result in extensive tissue destruction, even with the institution of appropriate antibiotic treatment, leading to reconstructive surgery and long-term disability. We report a case of a patient with severe oedematous BU, which describes for the first time the pre-emptive use of prednisolone therapy commenced at the time of antibiotic initiation aimed at limiting the ongoing tissue destruction and its secondary sequelae.

**Case presentation:**

A 91-year-old Australian-born Caucasian woman presented with a WHO category 3 oedematous BU lesion on the anterior aspect of her right ankle that she had first noticed three weeks earlier. Treatment was commenced with an antibiotic combination of rifampicin and ciprofloxacin. At the same time, pre-emptive prednisolone was commenced (a dose of 0.5mg/kg daily). Treatment resulted in rapid and significant reduction in the size of the induration associated with the lesion, and no significant increase in the size of the skin ulceration. Antibiotics were continued for 56 days and prednisolone therapy ceased 130 days after antibiotics commenced. No surgery was required. The wound healed completely after 10 months and there was no long-term limitation of movement at the ankle joint.

**Conclusions:**

Pre-emptive corticosteroid therapy may prevent further progressive tissue necrosis and the need for secondary reconstructive surgery that commonly occurs during the antibiotic treatment of severe odematous forms of BU.

## Introduction

*Mycobacterium ulcerans* causes destructive lesions of skin and subcutaneous tissue known as Buruli ulcers (BU). They are found in 33 countries worldwide but most commonly in west and central Africa and south-eastern Australia. Oedematous cases are the most severe form of disease, and often result in extensive tissue destruction leading to reconstructive surgery and long-term disability [1,2]. Combination antimicrobial therapy is now recommended as first-line treatment of BU [3,4] and results in high cure rates, limiting the requirements for surgery [5,6]. However, with oedematous lesions ongoing tissue destruction and increasing ulceration generally occurs despite the administration of appropriate antibiotics and limits the success of early antibiotics [1,7].

We report a case of a patient with severe oedematous BU and describe for the first time the pre-emptive use of prednisolone therapy commenced with the initiation of antibiotic treatment that appeared to limit ongoing tissue destruction and of the need for reconstructive surgery.

## Case presentation

**Figure 1:**
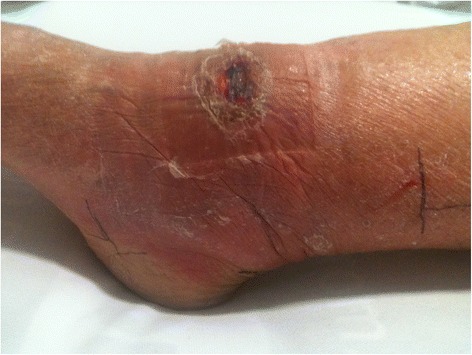
Severe oedematous Buruli ulcer lesion before antibiotic and prednisolone treatment. This figure shows the odematous Buruli ulcer lesion of the right ankle prior to treatment. There is a central area of developing necrosis anterior to the ankle joint, with a total area of induration associated with the lesion of 25.2cm^2^ (21cm diameter in a horizontal pane and 12cm diameter in a vertical plane). The black pen lines on the skin mark the edges of the induration in a vertical plane.

A 91-year-old previously healthy Australian-born Caucasian woman living in a BU endemic area in Victoria, Australia, presented with a three-week history of a painful lesion on the anterior aspect of her right ankle. On examination she had an indurated, oedematous lesion of 210 × 120mm in diameter with central necrosis of 20 × 10mm diameter, classified as a WHO category 3 lesion (Figure 1). She weighed 52kgs, and had an estimated glomerular filtration rate of approximately 60mLs/minute. A diagnosis of an oedematous BU lesion was made based on the clinical appearance of the lesion and a positive *M. ulcerans* PCR of a swab of the lesion.

She was commenced on rifampicin 450mg daily and ciprofloxacin 500mg twice daily. As we were concerned she was at risk of further tissue necrosis, pre-emptive prednisolone of 30mg daily (0.5mg/kg/day) was initiated at the same time.

**Figure 2:**
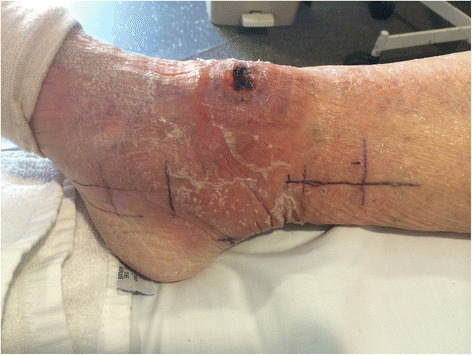
Severe oedematous Buruli ulcer lesion after three days of antibiotic and prednisolone treatment. This figure shows the odematous Buruli ulcer lesion of the right ankle three days after commencing antibiotics and prednisolone. The central area of ulceration remains unchanged in size, but the total area of induration associated with the lesion has reduced by 74% to 6.6cm^2^ (12cm diameter in a horizontal pane and 5.5cm diameter in a vertical plane). The black pen lines on the skin show the reduction in size of the induration in the vertical plane.

**Figure 3:**
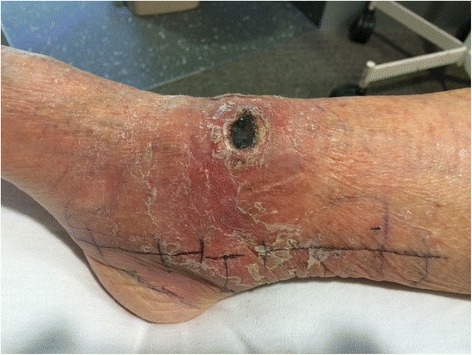
Severe oedematous Buruli ulcer lesion after six days of antibiotic and prednisolone treatment. This figure shows the odematous Buruli ulcer lesion of the right ankle six days after commencing antibiotics and prednisolone. The central area of ulceration remains unchanged in size, but the total area of induration associated with the lesion has reduced by 88% to 3.0cm^2^ (7.5cm diameter in a horizontal pane and 4.0cm diameter in a vertical plane). The black pen lines on the skin show the reduction in size of the induration in the vertical plane.

**Figure 4:**
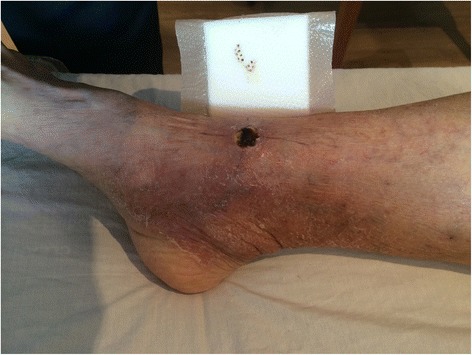
Severe oedematous Buruli ulcer lesion after one month of antibiotic and prednisolone treatment. This figure shows the odematous Buruli ulcer lesion of the right ankle one month after commencing antibiotics and prednisolone. It demonstrates only a small persisting area of ulceration over the ankle joint that has not increased significantly in size. The black pen lines on the skin that extend to the edges of the ulceration demonstrate that there is no induration associated with the lesion in the non-ulcerated skin.

After three days the induration associated with the lesion had reduced by 74% to 120 × 55mm in diameter and the size of the ulceration remained stable (Figure 2, Table 1). Further reduction in induration had occurred by day 6 (Figure 3) and after four weeks of prednisolone combined with antibiotics there was no induration associated with the lesion and the size of the ulceration remained stable (Figure 4).

**Table 1:** **Measurement of the area of skin induration and ulceration associated with the Buruli ulcer lesion according to the duration of treatment and dose of prednisolone**

**Days post start of treatment**	**Total induration (mm)**	**Total ulceration (mm)**	**Prednisolone dose (mg daily)**
0	210 × 120	20 × 10	30
3	120 × 55	20 × 10	30
6	75 × 40	20 × 11	30
10	55 × 30	20 × 14	20
26	No additional induration	20 × 18	10
31	No additional induration	20 × 15	10
38	No additional induration	20 × 16	5
44	NR	NR	Cease
58	No additional induration	17 × 11	0
83	60 × 60	17 × 11	5
102	No additional induration	20 × 10	5
109	NR	NR	2.5
116	No additional induration	20 × 10	2.5
130	No additional induration	19 × 12	Cease

NR, not reviewed.

The ciprofloxacin dose was reduced at day 3 to 250 mg twice daily because of nausea potentially caused by relatively high systemic ciprofloxacin levels related to her advanced age and moderately reduced renal function, and after 26 days of antibiotics she developed a rash and the ciprofloxacin was replaced by clarithromycin 250mg twice daily. Antibiotics were ceased after a total of 56 days. The prednisolone was well tolerated and was initially weaned off over 44 days. However, due to a subsequent increase in induration associated with the lesion, it was recommenced at a low dose (5mg daily) 83 days after antibiotics commenced, then weaned off 130 days after antibiotics commenced (Table 1). No surgical debridement or reconstruction was required.

**Figure 5:**
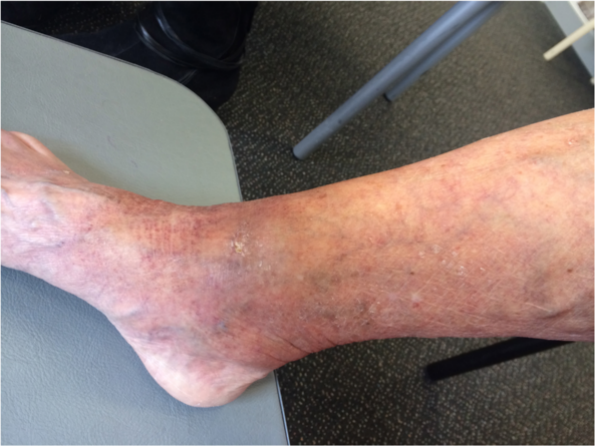
Severe oedematous Buruli ulcer lesion 10 months after commencing antibiotic and prednisolone treatment. This figure shows the odematous Buruli ulcer lesion of the right ankle 10 months after commencing antibiotics and prednisolone. The lesion has completely healed without any significant scar formation. There is a full range of movement at the ankle joint.

The lesion healed to a dry scab six months after commencing antibiotics, and had completely healed by 10 months (Figure 5). There was no permanent disability or limitation of movement at the ankle joint. There was no recurrence 12 months after treatment commenced.

## Discussion

Oedematous lesions account for about 7% of BU cases seen in the Bellarine Peninsula, South-Eastern Australia [1]. Compared to non-oedematous lesions they are more likely to occur in diabetics and those who have a lesion on the hand, elbow or ankle [1]. They represent the most aggressive form of BU with 76% of lesions in our health service presenting at an advanced stage (WHO category 2 or 3) due to rapid progression and delays in diagnosis [1]. As a result, oedematous BU lesions are often associated with significant tissue necrosis, and this can progress even after the commencement of appropriate antibiotic treatment [1,7]. Severe lesions usually require debridement and reconstructive surgery (94% in our experience) [1], increasing the cost of treatment [8] and often resulting in long-term disability [9].

Reasons for the ongoing ulceration despite appropriate antibiotic treatment are not known but may include tissue swelling and secondary ischaemia of skin and superficial tissues resulting from paradoxical reactions, analogous to the paradoxical enlargement and suppuration of tuberculous lymphadenitis on treatment [10]. These commonly occur during or after the antibiotic treatment of BU lesions [5,11], and are more frequent in the treatment of oedematous forms of the disease [1]. Alternatively, it may be due to a delayed effect of persisting mycolactone, a potent exotoxin produced by *M. ulcerans* that is toxic to tissues [12]. We have previously reported successful use of prednisolone to limit tissue damage in patients experiencing severe BU-associated paradoxical reactions without negative effects on curative outcomes [13,14]. Research from the mouse model also suggests corticosteroids could potentially be utilised in the treatment of paradoxical reactions without reducing the effectiveness of antibiotic treatment [15]. Therefore, if paradoxical reactions are the cause of ongoing tissue loss in oedematous BU lesions on antibiotic treatment, we have previously postulated that pre-emptive prednisolone may prevent it [1].

In this case, at the time of commencing antibiotic treatment, we started pre-emptive prednisolone at the dose recommended for treatment of BU-associated paradoxical reactions [3]. The aim was to try and minimize the progression of tissue necrosis, the need for reconstructive surgery and long-term potential complications such as limitation of movement of the ankle joint, which would likely have occurred in the absence of pre-emptive treatment.

We postulate that the pre-emptive prednisolone treatment was effective in achieving these aims with no progression of the ulceration on treatment, a rapid reduction in the size of the associated induration, the avoidance of surgery and the lesion healing completely with no long-term sequelae such as limitation of movement at the ankle joint. Prednisolone was well tolerated in this patient. However, it can have potential adverse effects that need to be considered before use such as the potential to worsen co-existent infections including hepatitis B, tuberculosis and strongyloides (of particular concern in west and central Africa where most BU cases occur), destabilise diabetes, cause gastrointestinal ulceration, and progression of osteoporosis. Therefore, we believe that further study, including prospective trials, be performed to assess the safety and efficacy of pre-emptive corticosteroid therapy in patients with severe oedematous BU.

In this case, we used the initial antibiotic combination rifampicin and ciprofloxacin, which we have found to be highly effective in curing BU lesions and preventing disease recurrences [6,16-18]. However, it should be noted that although this combination is one of the recommended first-line antibiotic regimens for BU treatment by Australian experts, WHO recommends the use of rifampicin and streptomycin as the standard antibiotic regimen [4].

## Conclusions

Our case suggests that pre-emptive corticosteroid therapy commenced at the time of antibiotic treatment initiation may prevent further progressive tissue necrosis and the need for secondary reconstructive surgery that commonly occurs during the antibiotic treatment of severe oedematous forms of BU.

## Consent

Written informed consent was obtained from the patient for publication of this case report and any accompanying images. A copy of the written consent is available for review by the Editor-in-Chief of this journal.

## References

[1] O'Brien DP, Friedman ND, McDonald A, Callan P, Hughes A, Athan E. Clinical features and risk factors of oedematous Mycobacterium ulcerans lesions in an Australian population: beware cellulitis in an endemic area. PLoS Negl Trop Dis. 2014;8, e2612. 10.1371/journal.pntd.0002612 PMC3879256 24392172

[2] Phanzu DM, Ablordey A, Imposo DB, Lefevre L, Mahema RL, Suykerbuyk P, et al. Short report: edematous Mycobacterium ulcerans infection (Buruli ulcer) on the face: a case report. Am J Trop Med Hyg. 2007;77:1099–102. 18165530

[3] O'Brien DP, Jenkin G, Buntine J, Steffen CM, McDonald A, Horne S, et al. Treatment and prevention of Mycobacterium ulcerans infection (Buruli ulcer) in Australia: guideline update. Med J Aust. 2014;200:267–70. 10.5694/mja13.11331 24641151

[4] World Health Organization. Treatment of Mycobacterium ulcerans disease (Buruli ulcer): guidance for health workers. Geneva, Switzerland: World Health Organization; 2012.

[5] Nienhuis WA, Stienstra Y, Thompson WA, Awuah PC, Abass KM, Tuah W, et al. Antimicrobial treatment for early, limited Mycobacterium ulcerans infection: a randomised controlled trial. Lancet. 2010;375:664–72. 10.1016/S0140-6736(09)61962-0 20137805

[6] Friedman ND, Athan E, Hughes AJ, Khajehnoori M, McDonald A, Callan P, et al. Mycobacterium ulcerans disease: experience with primary oral medical therapy in an Australian cohort. PLoS Negl Trop Dis. 2013;7, e2315. 10.1371/journal.pntd.0002315 PMC3715400 23875050

[7] Jenkin GA, Smith M, Fairley M, Johnson PD. Acute, oedematous Mycobacterium ulcerans infection in a farmer from far north Queensland. Med J Aust. 2002;176:180–1. 10.5694/j.1326-5377.2002.tb04350.x 11915879

[8] Pak J, O'Brien DP, Quek TY, Athan E. Treatment costs of Mycobacterium ulcerans in the antibiotic era. International Health. 2012;4:123–7. 10.1016/j.inhe.2011.12.005 24029150

[9] Stienstra Y, van Roest MH, van Wezel MJ, Wiersma IC, Hospers IC, Dijkstra PU, et al. Factors associated with functional limitations and subsequent employment or schooling in Buruli ulcer patients. Tropical Med Int Health. 2005;10:1251–7. 10.1111/j.1365-3156.2005.01519.x 16359405

[10] Carvalho AC, De Iaco G, Saleri N, Pini A, Capone S, Manfrin M, et al. Paradoxical reaction during tuberculosis treatment in HIV-seronegative patients. Clin Infect Dis. 2006;42:893–5. 10.1086/500459 16477577

[11] O'Brien DP, Robson M, Friedman ND, Walton A, McDonald A, Callan P, et al. Incidence, clinical spectrum, diagnostic features, treatment and predictors of paradoxical reactions during antibiotic treatment of Mycobacterium ulcerans infections. BMC Infect Dis. 2013;13:416. 10.1186/1471-2334-13-416 PMC3854792 24007371

[12] Guenin-Mace L, Veyron-Churlet R, Thoulouze MI, Romet-Lemonne G, Hong H, Leadlay PF, et al. Mycolactone activation of Wiskott-Aldrich syndrome proteins underpins Buruli ulcer formation. J Clin Invest. 2013;123:1501–12. 10.1172/JCI66576 PMC3613928 23549080

[13] Friedman ND, McDonald A, Robson M, O'Brien DP. Corticosteroid use for paradoxical reactions during antibiotic treatment for Mycobacterium ulcerans. PLoS Negl Trop Dis. 2012;6, e1767. 10.1371/journal.pntd.0001767 PMC3459890 23029568

[14] Wanda F, Nkemenang P, Ehounou G, Tchaton M, Comte E, Toutous Trellu L, et al. Clinical features and management of a severe paradoxical reaction associated with combined treatment of Buruli ulcer and HIV co-infection. BMC Infect Dis. 2014;14:423. 10.1186/1471-2334-14-423 PMC4122778 25073531

[15] Martins TG, Trigo G, Fraga AG, Gama JB, Longatto-Filho A, Saraiva M, et al. Corticosteroid-induced immunosuppression ultimately does not compromise the efficacy of antibiotherapy in murine Mycobacterium ulcerans infection. PLoS Negl Trop Dis. 2012;6, e1925. 10.1371/journal.pntd.0001925 PMC3510087 23209864

[16] O'Brien DP, Hughes AJ, Cheng AC, Henry MJ, Callan P, McDonald A, et al. Outcomes for Mycobacterium ulcerans infection with combined surgery and antibiotic therapy: findings from a south-eastern Australian case series. Med J Aust. 2007;186:58–61. 10.5694/j.1326-5377.2007.tb00800.x 17223763

[17] O'Brien DP, Athan E, Hughes A, Johnson PD. Successful treatment of Mycobacterium ulcerans osteomyelitis with minor surgical debridement and prolonged rifampicin and ciprofloxacin therapy: a case report. J Med Case Rep. 2008;2:123. 10.1186/1752-1947-2-123 PMC2396654 18439310

[18] O'Brien DP, McDonald A, Callan P, Robson M, Friedman ND, Hughes A, et al. Successful outcomes with oral fluoroquinolones combined with rifampicin in the treatment of Mycobacterium ulcerans: an observational cohort study. PLoS Negl Trop Dis. 2012;6, e1473. 10.1371/journal.pntd.0001473 PMC3260310 22272368

